# Improvements following multimodal pelvic floor physical therapy in gynecological cancer survivors suffering from pain during sexual intercourse: Results from a one-year follow-up mixed-method study

**DOI:** 10.1371/journal.pone.0262844

**Published:** 2022-01-25

**Authors:** Marie-Pierre Cyr, Rosalie Dostie, Chantal Camden, Chantale Dumoulin, Paul Bessette, Annick Pina, Walter Henry Gotlieb, Korine Lapointe-Milot, Marie-Hélène Mayrand, Mélanie Morin

**Affiliations:** 1 Faculty of Medicine and Health Sciences, School of Rehabilitation, University of Sherbrooke, Sherbrooke, Quebec, Canada; 2 Research Center of the Centre Hospitalier Universitaire de Sherbrooke, Sherbrooke, Quebec, Canada; 3 Faculty of Medicine, School of Rehabilitation, University of Montreal, Montreal, Quebec, Canada; 4 Research Center of the Institut Universitaire de Gériatrie de Montréal, Montreal, Quebec, Canada; 5 Faculty of Medicine and Health Sciences, Division of Gynecologic Oncology, Department of Obstetrics and Gynecology, University of Sherbrooke, Sherbrooke, Quebec, Canada; 6 Faculty of Medicine, Division of Gynecologic Oncology, Department of Obstetrics and Gynecology, University of Montreal, Montreal, Quebec, Canada; 7 Research Center of the Centre Hospitalier de l’Université de Montréal, Montreal, Quebec, Canada; 8 Faculty of Medicine, Division of Gynecologic Oncology, Department of Obstetrics and Gynecology, McGill University, Montreal, Quebec, Canada; 9 Lady Davis Institute of the Jewish General Hospital, Montreal, Quebec, Canada; 10 Faculty of Medicine, Departments of Obstetrics and Gynecology and Social and Preventive Medicine, University of Montreal, Montreal, Quebec, Canada; Dipartimento di Scienze Mediche e Chirugiche (DIMEC), Orsola Hospital, ITALY

## Abstract

**Background:**

A large proportion of gynecological cancer survivors suffer from pain during sexual intercourse, also known as dyspareunia. Following a multimodal pelvic floor physical therapy (PFPT) treatment, a reduction in pain and improvement in psychosexual outcomes were found in the short term, but no study thus far has examined whether these changes are sustained over time.

**Purpose:**

To examine the improvements in pain, sexual functioning, sexual distress, body image concerns, pain anxiety, pain catastrophizing, painful intercourse self-efficacy, depressive symptoms and pelvic floor disorder symptoms in gynecological cancer survivors with dyspareunia after PFPT, and to explore women’s perceptions of treatment effects at one-year follow-up.

**Methods:**

This mixed-method study included 31 gynecological cancer survivors affected by dyspareunia. The women completed a 12-week PFPT treatment comprising education, manual therapy and pelvic floor muscle exercises. Quantitative data were collected using validated questionnaires at baseline, post-treatment and one-year follow-up. As for qualitative data, semi-structured interviews were conducted at one-year follow-up to better understand women’s perception and experience of treatment effects.

**Results:**

Significant improvements were found from baseline to one-year follow-up on all quantitative outcomes (*P* ≤ 0.028). Moreover, no changes were found from post-treatment to one-year follow-up, supporting that the improvements were sustained at follow-up. Qualitative data highlighted that reduction in pain, improvement in sexual functioning and reduction in urinary symptoms were the most meaningful effects perceived by participants. Women expressed that these effects resulted from positive biological, psychological and social changes attributable to multimodal PFPT. Adherence was also perceived to influence treatment outcomes.

**Conclusions:**

Findings suggest that the short-term improvements following multimodal PFPT are sustained and meaningful for gynecological cancer survivors with dyspareunia one year after treatment.

## Introduction

An increasing number of women live with the deleterious, long-term consequences of cancer [1,2]. Alongside urinary incontinence, chronic pain during sexual intercourse, also known as dyspareunia, is one of the most common sexual health issues, affecting more than half of gynecological cancer survivors [3,4]. Dyspareunia is recognized as resulting from the complex interaction of anatomical, physiological, psychological and relationship factors related to cancer and oncological treatments [5], in line with the biopsychosocial model [6,7]. Vaginal stenosis, impaired tissue flexibility, heightened pelvic floor muscle tone and contractility impairments as well as vaginal dryness [5,8] may contribute to experiencing pain during intercourse. These biological factors interplay with pain anxiety (i.e., fear of pain), pain catastrophizing [9] and low pain self-efficacy [10], thereby intensifying the pain [11]. Gynecological cancer survivors are also at risk of depressive symptoms and body image concerns [12,13], which may disturb how they perceive themselves as women [14–16]. These pain and psychological factors may contribute to sexual distress [17,18]. Moreover, women who have been treated for gynecological cancer are often affected by other sexual dysfunctions such as loss of libido or sexual desire [17]. All this can lead to relationship difficulties [12,13], disrupting their quality of life [19–21].

Despite the high prevalence of dyspareunia, there are limited treatment options supported by empirical evidence. Clinical survivorship guidelines suggest multimodal pelvic floor physical therapy (PFPT) as a nonhormonal, non-pharmacological and non-invasive first-line treatment to alleviate dyspareunia in cancer survivors [22–24]. Through psychosexual education, manual therapy techniques and pelvic floor muscle exercises, PFPT targets the consequences of oncological treatments by restoring the pelvic floor tissues [8] while providing support and guidance to women to resume painless sexual activities [25,26]. So far, only one recent multicenter prospective study conducted by our team investigated a 12-week PFPT treatment in this population [27]. Significant changes in biological and psychosexual outcomes were found following treatment [27–29]. Using a comprehensive assessment combining intra-vaginal dynamometry and ultrasound imaging, pelvic floor muscle tone was significantly reduced while tissue flexibility, muscle contractile properties, control as well as endurance significantly improved immediately after treatment [28]. An increase in vaginal dimensions and a reduction in vaginal atrophy signs were also measured [28]. Concurrently, pain during intercourse, sexual distress, body image concerns, pain anxiety, pain catastrophizing, depressive symptoms, urinary symptoms, vaginal symptoms and sexual matters decreased while sexual functioning and pain self-efficacy improved after PFPT [27,29].

To date, no study has examined whether the short-term improvements following PFPT in gynecological cancer survivors with dyspareunia are sustained over time. Long-term treatment effects have important socioeconomic implications [30,31], and evaluating them may provide critical insights beyond those assessed in the short term [32]. More importantly, using only quantitative methods may not be sufficient to fully capture the extent of PFPT effects as these are multidimensional and likely depend on the interaction of multiple factors [6]. Furthermore, it has been recently recognized that PFPT is not only a physical treatment but it is also a behavioral treatment, which emphasizes the relevance of investigating physical, cognitive and behavioral outcomes associated with PFPT [33]. Combining quantitative and qualitative methods would therefore provide a better understanding of the treatment effects and how they influence each other considering the clinical context of multimodal PFPT [34,35]. This mixed-method study aimed to examine the improvements in pain, sexual functioning, sexual distress, body image concerns, pain anxiety, pain catastrophizing, pain self-efficacy, depressive symptoms and pelvic floor disorder symptoms in gynecological cancer survivors with dyspareunia after multimodal PFPT, and to explore women’s perceptions of treatment effects at one-year follow-up.

## Materials and methods

### Design and methodology

This study is a planned follow-up study of a multicenter prospective interventional study investigating the treatment effects of multimodal PFPT for gynecological cancer survivors with dyspareunia [27]. Our intent was to follow the whole cohort instead of a subsample in order to most closely match the primary trial (mainly in terms of participant characteristics and study outcomes) [32]. This research was conducted in Sherbrooke and Montreal (Canada). Changes from baseline to post-treatment have been published elsewhere [27–29], and changes from baseline and post-treatment to one-year follow-up will be the focus of the present manuscript. The participants underwent baseline, post-treatment and one-year follow-up assessments. Quantitative data were collected at all time points. To ascertain and advance our understanding of treatment effects at one-year follow-up, individual semi-structured telephone interviews were carried out to collect qualitative data [34,35]. This research was approved by the Ethics Review Board of the CIUSSS de l’Estrie–CHUS (MP-31-2016-1322) and was registered on ClinicalTrials.gov (NCT03935698). Participants provided written informed consent.

### Participants

Women were included according to the following criteria: (i) all planned oncological treatments for either endometrial or cervical cancer (stages ranging from I to IV) completed for at least three months; (ii) in remission given the absence of disease on radiologic imaging for at least three months; (iii) moderate to severe vulvovaginal pain during sexual intercourse (i.e., pain at the entry of the vagina and at the mid-vagina, at the level of the pelvic floor muscles), corresponding to a pain intensity of 5 or more on a Numerical Rating Scale (NRS) ranging from 0 (no pain) to 10 (worst pain); (iv) vulvovaginal pain experienced in more than 80% of sexual intercourse for at least three months; (v) a stable sexual partner; and (vi) willingness to attempt vaginal penetrations. A gynecologic oncologist of our team at each site performed a standardized gynecological examination to rule out other conditions possibly causing dyspareunia (e.g., vaginitis, cystitis or dermatitis). Exclusion criteria were: (i) inability to communicate in French or English; (ii) dyspareunia prior to cancer or pelvic pain unrelated to intercourse; (iii) other pelvic conditions including urinary tract or vaginal infection, deep pelvic pain (i.e., pain experienced in the abdomen with deep penetration), chronic constipation, severe pelvic organ descent based on the Pelvic Organ Prolapse–Quantification system (stage III or more); (iv) other primary pelvic cancer or breast cancer; (v) any history of vulvar, vaginal or pelvic surgery unrelated to cancer; (vi) PFPT in the last year; (vii) changes in the use or dosage of menopausal hormone therapy in the last six months; (viii) a major medical or psychological condition likely to interfere with study procedures; or (ix) refusal to abstain from using other treatments for dyspareunia until the post-treatment assessment.

### Treatment content

The treatment protocol was designed by a multidisciplinary team consisting of experts in gynecologic oncology, physical therapy, psychology and sexual health. The treatment included 12 weekly sessions of 60 minutes with a physical therapist certified and experienced in pelvic and women’s health. The treatment components were chosen to reflect practice in a clinical setting [36]. At each session, the physical therapist provided information, advice and support to women. She explained the underlying mechanisms of chronic pain experienced during sexual intercourse after gynecological cancer including the role of the pelvic floor muscles and how the treatment could help to reduce the pain. She gave additional information about how to manage chronic pain and other pelvic floor disorder symptoms (e.g., bladder training). The use of relaxation techniques using deep breathing as well as the application of vaginal lubricants and moisturizers were encouraged. The physical therapist also helped the participants gain more knowledge about sexual functioning (i.e., physiology of desire, excitation and orgasm) and guided them into resuming non-painful sexual activities with their partners. The latter was invited to participate in the treatment to help his partner in this process. Moreover, the physical therapist was available to further discuss topics with the participants who were invited to reflect on their sexual difficulties in order to overcome them with the help of their therapist. At each session, manual therapy techniques (i.e., stretching, myofascial release and tissue desensitization) and pelvic floor muscle exercises with electromyography biofeedback (i.e., relaxation, motor control, strength and endurance) using a small intra-vaginal probe were used. Women were also asked to perform home exercises resembling those performed under supervision five times per week as well as auto-insertion exercises with a finger or graded vaginal dilator in addition to desensitization techniques three times per week. Throughout the treatment, the physical therapist supervised each woman’s progress and provided feedback. Additionally, modalities were intensified (e.g., more pressure applied to stretch the tissues, longer duration of the technique or exercise and greater dilator size) following each woman’s progress. At the end of the treatment, women were encouraged to pursue home exercises two to three times per week to maintain the effects of treatment. Further details of the treatment modalities are presented elsewhere [27].

### Data collection

Participants were assessed at baseline, post-treatment and one-year follow-up. Sample characteristics were collected at baseline. At each time point, quantitative outcomes were assessed using validated scales and questionnaires. After the collection of quantitative data at one-year follow-up, an individual semi-structured telephone interview was conducted in French or in English to further explore women’s perceptions of treatment effects. Participants were also asked if there were any changes regarding their health (e.g., cancer recurrence), if they were pursuing the home exercises, if they had attempted other treatments for pain or sexual dysfunction and if their relationship status had changed during the follow-up period.

### Study outcomes

#### Quantitative

The NRS was used to evaluate the average intensity of pain during intercourse [37]. The McGill Pain Questionnaire (MPQ) was used to qualify the pain according to its sensory, affective and evaluative dimensions, with higher scores corresponding to more significant pain [38]. The Female Sexual Function Index (FSFI) was used to examine sexual functioning including desire, arousal, lubrication, orgasm, satisfaction and pain, with higher total scores representing a better sexual function [39,40]. The Female Sexual Distress Scale-Revised (FSDS-R) was used to assess sexual distress, with higher scores relating to more sexual distress [41,42]. The Body Image Scale (BIS) was administered to evaluate body image concerns, with higher scores indicating greater concerns [43]. The Pain Anxiety Symptom Scale (PASS), which is an indirect measure of fear of pain during intercourse, was used to assess pain-related anxiety, with higher scores indicating more severe pain anxiety [44]. The Pain Catastrophizing Scale (PCS) was used to evaluate the exaggerated negative cognitions and emotions regarding pain, with higher scores pointing to greater pain catastrophizing [45]. The Painful Intercourse Self-Efficacy Scale (PISES) was used to assess pain self-efficacy associated with painful sexual intercourse, with higher scores representing better self-efficacy [46]. The Beck Depression Inventory-II (BDI-II) was used to evaluate depressive symptoms, with higher scores corresponding to higher severity of symptoms [37]. Pelvic floor disorder symptoms including urinary symptoms, vaginal symptoms and sexual matters were assessed with the International Consultation on Incontinence Questionnaire (ICIQ) modules. The ICIQ-Urinary Incontinence Short Form (ICIQ-UI SF) was used for urinary symptoms [47] and the ICIQ-Vaginal Symptoms (ICIQ-VS) for vaginal symptoms and sexual matters [48], with higher scores representing more symptoms or sexual matters [47,48]. In addition, the Patient Global Impression of Change (PGIC) allowed the participants to self-report their perceived improvement (categories ranging from very much improved to very much worse) [49].

#### Qualitative

Prior to their individual semi-structured telephone interview, participants were informed of the interview topics and invited to reflect on the treatment effects they perceived and how these effects evolved over time during the follow-up period. Each interview lasted approximately 70 minutes. The first author (MPC) underwent qualitative research training to conduct all the interviews. She was not involved in participants’ care and was blinded to the participants’ responses in the questionnaires to avoid any preconceived ideas about the treatment effects. Before conducting the interviews, the interviewer reconfirmed the women’s consent to participate in the interviews and for recording the conversation. She used a nonjudgmental approach and created a trustful and respectful relationship to ease the discussion of what could be perceived by participants as sensitive topics. Interviews followed a semi-structured guide co-constructed by the first author (MPC), the principal investigator (MM) and another research team member who has extensive experience conducting qualitative research (CC) (see S1 File for the interview guide). The interview questions related to this manuscript’s research objective focused on women’s perceptions of treatment effects and their hypotheses about factors influencing these effects. Probing questions aimed to obtain in-depth information about participants’ perceptions of treatment effects, exploring short-term effects previously documented in quantitative research [27–29] and using a biopsychosocial approach of health to explore any further effects and factors perceived to influence these effects [6,7]. The semi-structured guide was pilot-tested with a patient partner under the supervision of the principal investigator (MM) and the other research team member (CC).

### Sample size

An *a priori* sample size was calculated for the multicenter prospective interventional study based on the proportion of completed home exercises (80%) as adherence was suggested as being important to perceive significant effects in physical therapy [50]. With a confidence level of 95%, an interval width of 30%, and to account for potential dropouts over time (15%), a total of 31 women were initially recruited for quantitative purposes (further details are available elsewhere) [27]. All these women were invited to take part in an individual semi-structured telephone interview to explore all of the various perceptions of treatment effects.

### Data analysis

Quantitative data analysis was performed using IBM SPSS Statistics 27 (IBM Corporation, Armonk, N.Y., USA). Descriptive statistics were used to present baseline and one-year sample characteristics as well as PGIC results. Intention-to-treat analyses (i.e., all participants are included in the statistical analysis, regardless of their level of adherence) were conducted to explore whether the improvements in all outcomes were sustained at one-year follow-up. Outcomes at baseline and one-year follow-up as well as the changes from baseline and post-treatment to one-year follow-up are reported and expressed as mean estimated values (95% confidence interval) according to linear mixed modeling with Bonferroni correction [51–53]. Models included time as the fixed effect and random intercepts for each subject to account for repeated measures (i.e., to accommodate within-subject correlation). Statistical significance was set at p-value < 0.05 (two-tailed).

Qualitative data analysis was based on the audio-recorded interviews which were transcribed and analyzed by the first author (MPC) using NVivo (version 12) software. A thematic analysis was adopted to ensure data-driven analyses and interpretations [54]. Specifically, an inductive approach was used when the first author (MPC) coded key ideas and started identifying emerging themes. Subsequently, another team member (RD) reviewed the codes. Coding disagreements were discussed until a consensus was achieved. Codes were reviewed by two research team members (MM and CC), and several meetings were held to regroup codes into themes. Relationships between themes were explored by observing patterns across themes. As most of the original quotations used in this manuscript were in French, they were translated into English and revised by a certified translator. Field notes were used to explore researcher reflexivity and further support the data interpretation. It should be noted that results from quantitative and qualitative methods were integrated during the interpretation phase of the study.

## Results

### Participant characteristics

**Fig 1:**
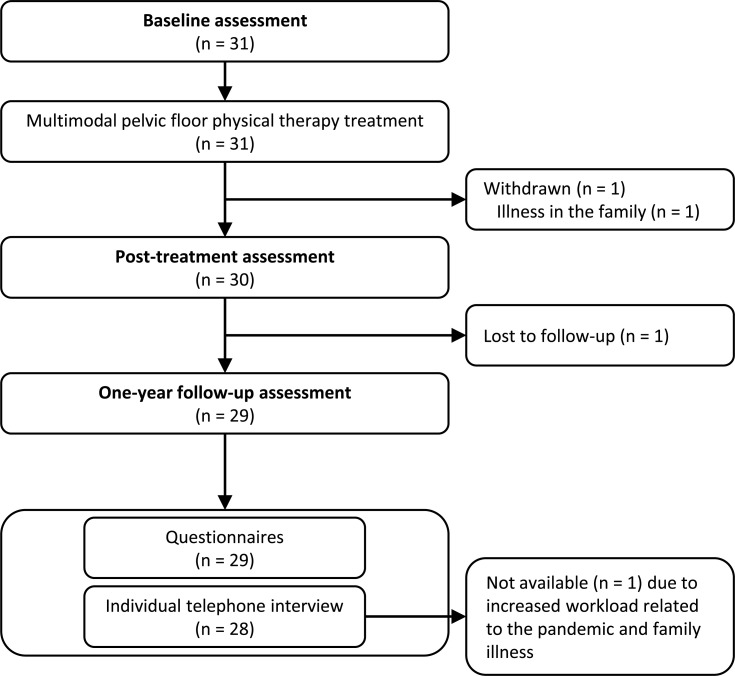
Flow of participants through the study.

Thirty-one women enrolled initially in this study. Fig 1 shows the flow of participants through the study. Additional details on screening and eligibility assessments are available elsewhere [27].

Baseline sample characteristics (n = 31) are presented in Table 1. Before the multimodal PFPT treatment, women had an average pain intensity of 7.3 (6.7 to 8.0) on the NRS and the median duration of pain was approximately three years. Of the 29 women assessed at one-year follow-up, three reported having had a cancer recurrence or another cancer during the follow-up period, and one was recovering from a severe upper urinary tract infection.

**Table 1:** Sample characteristics at baseline.

Characteristics	Value
Age (years), mean (SD)	55.9 (10.8)
Body mass index (kg/m^2^), mean (SD)	28.5 (5.3)
Cancer type, n (%)	
Endometrial	20 (64.5)
Cervical	11 (35.5)
Disease stage, n (%)	
I	19 (61)
II	6 (19)
III	5 (16)
IV	1 (3)
Time since oncological treatments (months), median (Q1 to Q3)	38 (9 to 70)
Oncological treatments, n (%)	
Surgery alone	9 (29)
Surgery + brachytherapy or external beam radiation therapy	6 (19)
Surgery + brachytherapy + external beam radiation therapy + chemotherapy	7 (23)
Surgery + chemotherapy	2 (6)
Brachytherapy + external beam radiation therapy + chemotherapy	7 (23)

SD, standard deviation; n, number of participants; Q1, first quartile; Q3, third quartile.

### Study outcomes

#### Quantitative

The quantitative outcomes assessed at baseline and one-year follow-up as well as the changes from baseline and post-treatment to one-year follow-up are presented in Table 2. Significant improvements were found from baseline to one-year follow-up on all outcomes (*P* ≤ 0.028). Moreover, changes from post-treatment to one-year follow-up were statistically non-significant (*P* ≥ 0.084), suggesting that the improvements were maintained over time. Of the 29 women assessed at one-year follow-up, 25 (86%) reported being very much or much improved. The others reported minimal improvements (7%), no changes (3%) or being minimally worse (3%) compared to baseline. Concerning the adherence to home exercises, 18 (62%) performed the pelvic floor muscle exercises during the follow-up period, with a median frequency of three times (two to eight) per month. Moreover, 10 (34%) participants performed the auto-insertion exercises, with a median frequency of three times (one to five) per month. No women stated having attempted other treatments for pain or sexual dysfunction during this period, and only one reported being no longer with her partner at one-year follow-up.

**Table 2:** Outcomes at baseline (n = 31) and one-year follow-up (n = 29) and changes from baseline and post-treatment to one-year follow-up.

	Baseline	One-year follow-up	Changes from baseline to follow-up	*P* ^a^	Changes from post-treatment to follow-up	*P* ^a^
**Pain intensity** NRS (0–10)	7.3 (6.7 to 8.0)	2.7 (2.0 to 3.3)	-4.6 (-5.7 to -3.6)	< 0.001	1.0 (-0.1 to 2.0)	0.084
**Pain quality** MPQ (0–78)	21.1 (17.6 to 24.6)	6.7 (3.1 to 10.4)	-14.4 (-20.5 to -8.3)	< 0.001	-0.5 (-6.7 to 5.6)	1.000
**Sexual function** FSFI (2–36)	18.9 (16.3 to 21.4) (n = 20)^b^	23.4 (20.8 to 26.0) (n = 18)^b^	4.6 (1.0 to 8.1)	0.009	-2.8 (-6.2 to 0.5)	0.119
**Sexual distress** FSDS-R (0–52)	26.7 (22.3 to 31.1)	16.6 (12.1 to 21.1)	-10.0 (-15.7 to -4.4)	< 0.001	2.7 (-2.9 to 8.4)	0.708
**Body image concerns** BIS (0–30)	6.4 (4.8 to 7.9)	3.0 (1.4 to 4.6)	-3.4 (-5.4 to -1.3)	< 0.001	0.1 (-1.9 to 2.1)	1.000
**Pain anxiety** PASS (0–100)	37.5 (32.4 to 42.7)	23.7 (18.4 to 28.9)	-13.9 (-21.6 to -6.2)	< 0.001	2.8 (-5.0 to 10.5)	1.000
**Pain catastrophizing** PCS (0–52)	20.9 (16.6 to 25.2)	8.3 (3.9 to 12.7)	-12.6 (-18.1 to -7.1)	< 0.001	0.6 (-5.0 to 6.1)	1.000
**Painful intercourse self-efficacy** PISES (10–100)	63.6 (58.1 to 69.0)	80.6 (75.0 to 86.2)	17.1 (10.1 to 24.1)	< 0.001	-6.3 (-13.4 to 0.7)	0.095
**Depressive symptoms** BDI-II (0–63)	10.9 (8.0 to 13.9)	7.5 (4.5 to 10.5)	-3.5 (-6.6 to -0.3)	0.028	1.1 (-2.1 to 4.2)	1.000
**Urinary symptoms** ICIQ-UI (0–21)	3.8 (2.5 to 5.2)	1.8 (0.4 to 3.3)	-2.0 (-3.3 to -0.6)	0.002	-0.5 (-1.8 to 0.9)	1.000
**Vaginal symptoms** ICIQ-VS (0–53)	13.5 (11.5 to 15.4)	7.2 (5.2 to 9.2)	-6.3 (-8.6 to -4.0)	< 0.001	-0.4 (-2.7 to 1.9)	1.000
**Sexual matters** ICIQ-VS (0–58)	43.7 (37.7 to 49.7) (n = 24)^c^	20.9 (14.8 to 27.0) (n = 23)^c^	-22.8 (-32.3 to -13.4)	< 0.001	1.2 (-8.0 to 10.3)	1.000

The data shown are the mean estimated values (95% confidence interval) derived from the linear mixed models.

^a^ *P*-values extracted from the linear mixed modeling with Bonferroni correction.

^b^ Eleven women at baseline and 11 women at one-year follow-up did not engage in sexual activities including vaginal penetration in the last month and thereby, due to the one-month time frame used in the FSFI questionnaire, their total score could not be compilated. Reasons for not engaging in such activities at one-year follow-up: 4 = partner-related reasons including lack of sexual desire or medical problems such as erectile problems; 4 = participant-related reasons including lack of sexual desire (n = 2) or pain during intercourse (n = 2) although they reported a pain reduction of 4.5 and 5 on the NRS from baseline to one-year follow-up; 2 = relationship-related difficulties; 1 = medical indication to not engage due to vaginal bleeding unrelated to PFPT.

^c^ Seven participants at baseline and six at one-year follow-up did not engage in any form of sexual activities in the last month (time frame of ICIQ-VS for sexual matters).

#### Qualitative

**Fig 2:**
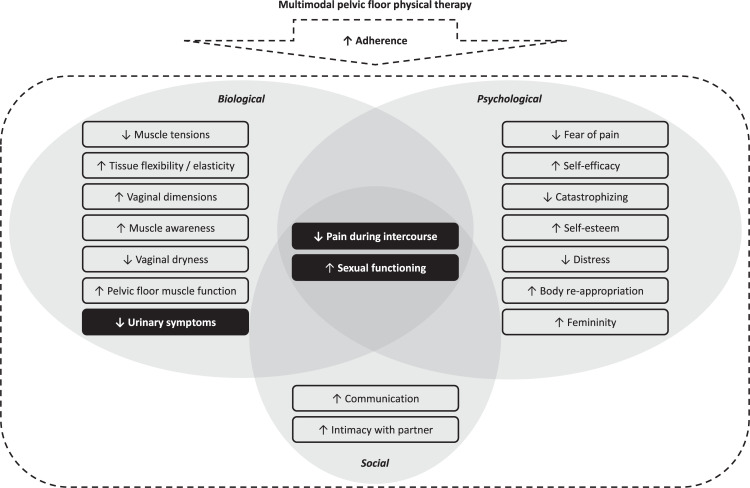
Relationships between treatment effects that emerged from the interviews.

Three main themes were described by participants as the most meaningful treatment effects for them in terms of symptoms or functioning: (a) reduction in pain during intercourse; (b) improvement in sexual functioning; and (c) reduction in urinary symptoms. These themes are detailed below along with participants’ perceived modulating and contributing factors. Modulating factors were defined as the factors altering the magnitude of the main effects (e.g., adherence) while contributing factors were those described as other treatment effects which influenced positively the main effects (e.g., reduction in muscle tensions). Fig 2 illustrates how the main treatment effects (in black) interacted and were influenced by various biological, psychological or social factors (in grey).

*THEME 1*. *Reduction in pain during intercourse*. All participants reported experiencing less pain during intercourse, with several stating having no pain at all since the end of the PFPT treatment. Although the majority expressed that this effect was maintained, a small number of women said that the pain reduction was attenuated at one-year follow-up. Among the potential explanations, some of them suggested that discontinuing home exercises or stopping regular sexual intercourse with vaginal penetration might have contributed to this depletion effect.

*“It fixed my pain problem and it lasted over time.”–C02*

*“I would say that it has deteriorated a bit since, but it’s my fault because I didn’t keep doing the exercises long enough. I know if I resumed the exercises it would get better. However, it [the pain] hasn’t come back to how it was before; in other words what has been done has been of benefit. Having sexual intercourse regularly helps to ensure these gains are maintained in a way.”–C12*

Every participant associated the pain reduction with pelvic floor tissue changes. They noticed that the muscle tensions decreased while the tissue flexibility increased, attributing this to the manual techniques and the exercises. Some emphasized that relaxation techniques such as deep breathing promoted muscle relaxation, reduction of tensions, and hence, a pain relief. Overall, the women related these tissue changes to a less tense or deeper vagina, which allowed them to be more at ease and helped them to have a more complete and comfortable vaginal penetration with less or no pain.

*“All the exercises [contraction and stretching] I had done and what the physical therapist had done removed the tension and loosened me up. It felt good. Penetration was easier.”–C01*

*“The stretching we did reduced my pain because when it stretches better, it’s less painful. Otherwise, I felt like the skin inside wanted so badly to split because, before, it wouldn’t stretch.”–C18*

*“Breathing helps because I think when you calm down, it’s less contracted and there’s more flexibility for the activity.”–C16*

Many women also observed becoming more aware of the pelvic floor musculature and its relationship with pain. During the PFPT treatment, they recalled gaining control over their muscles and developing muscle awareness. Motor control was noted as being important by the participants to break a chain of events involving the pelvic floor muscles and pain.

*“When you are calmer, it [the pelvic floor muscle] is less contracted, so it is more flexible. […] Before the treatments, I didn’t know how to do [relax my muscles], I was tense. Now, I have techniques that last over time. […] I have gone from… not hysteria, but from an uncontrolled fear to something more serene. I am calmer when considering having sex, I am more welcoming.”–C16*

Our participants often mentioned being reassured knowing how to influence the pain. They frequently expressed being less afraid of pain because they understood what led to their symptoms and were taught relevant and effective tools to reduce it.

*“After cancer treatments, you feel diminished. Will it come back as before? I was starting to be afraid. With physical therapy, you feel less diminished. It seemed as if it was finally possible that things could get better. When I got into the program, it was another story as I realized it was possible to improve, and it was much less upsetting, less scary. It’s because we found where it hurt most. It’s about understanding… It’s partly confidence, partly the fear that’s gone.”–C124*

Consequently, they explained that they were feeling more in control, self-efficient and hopeful while being less anxious about their pain. Some participants even emphasized that they were no longer afraid to undergo gynecological examinations. Experiencing less pain during intercourse also enhanced these feelings, which in turn amplified their self-esteem and confidence to engage in sexual activities. They felt less distressed, with several highlighting that fact they were less depressed and more positive in their everyday lives.

*“And what I also learned was that I felt that I could influence my pain. When it’s less painful, less tight, you are more relaxed, you have more confidence and you let go more easily. Psychologically, I could say that I felt I was moving further away from the operation and its negative side. I found that I was getting closer to a more normal life, as it was before, in a sense… without much difficulty. Yes, it’s vague, isn’t it? Well, normal life… having sex again, get away from the cancer thing.”–C115*

*THEME 2*. *Improvement in sexual functioning*. All women reported improvement in their sexual functioning following PFPT. Although a low proportion of participants did not perceive changes in their sexual functioning in terms of lubrication and libido or sexual desire, the vast majority mentioned their vagina being less dry and more naturally lubricated during sexual activities. Among other things, several women emphasized not needing to use vaginal products anymore and reported being less stressed and more interested in engaging in sexual activities.

*“The lubrication… it all came basically together after the treatment. Sure, at first I needed some lubricant, but little by little, as I worked, it just faded so I didn’t need the lubricant anymore.”–C09*

The perceptions relating to pain reduction described previously could also suggest how participants felt about sexuality. Many of them reported being more interested in engaging given the pain reduction and the positive emotions and thoughts they developed about their sexual identity. Some women associated their increased sexual desire to the improved perception of their body, which defined them as women. They grew to accept themselves, sensed that their body belonged to them and reclaimed it. Participants specified that this body re-appropriation helped them to express themselves sexually as women. They were able to have sexual intercourse with vaginal penetration rather than endure the barriers induced by cancer, which hampered them. Consequently, they referred to being complete women and having a more normal life. Participants related that regaining the capacity of having intercourse helped them initiate and engage in sexual activities, which in turn increased their femininity.

*“I could see that there were still defects in my body since the operation and all that, and psychologically it disturbed me. Now, I let myself go more. There is a connection that has been made with my body and my whole person. I participate more with my body now, which I didn’t before. I had an easier time opening up to sexuality. That’s why I say it really… changed my life. Physical therapy is beneficial, it is a psycho-unblocker.”–C100*

*“Knowing what to do to have intercourse and being able to have it [sexual intercourse] really made me feel like a woman. I am very happy to have learned to control my body better and to be able to have a more fulfilling sex life. It’s like… I feel like more of a complete woman, I don’t know… entirely a woman.”–C11*

*“Basically, sexuality is more about being a complete woman, […] Now, if I feel like having sex, I can have it. […] So, life for me is much more normal than it used to be. It changed my life, it gave me back intimacy. So, we’re less active than we were, but at least if we want to, we can! So that’s the difference.”–C10*

Participants also recognized that they were more comfortable talking about sexuality. They stressed that this led them to communicate more about their feelings and difficulties to their partner. As a result, participants and their partners were more capable of adapting their behavior, and when considering physical intimacy, it was therefore less stressful and more pleasurable. Furthermore, participants said that because they had less pain during intercourse, their partner was less afraid to hurt them, and this dynamic was helpful for the couple to be physically intimate.

*“I was also able to talk about it [thoughts about intercourse] with my partner because I had not talked about it before. When I had intercourse before, it was because I felt obliged. It was very rare that we had any. With the study, it was like day and night, winter and summer. It was like having sex two or three times a week by the end of the study.”–C06*

*“I was no longer in pain… well, for sure in our intimate relationship and all that there was a letting go so that was really amazing. Less fear, less apprehension. Yes, I think it reassured my husband a lot to see that it was going well, that it was getting better. He was also less afraid of hurting me and he was more reassured that there were two of us in this sexual activity.”–C08*

Because they were more communicative, most women acknowledged that they and their partner discussed their sexuality and intimacy more openly. Those who did not report any changes in this regard claimed their relationship was already strong and without issues before enrolling in the study. The former noticed that they and their partner were closer to each other, discovered and tried new ways to express their love. Several participants spoke of how it became more affectionate than sexually demonstrative with intercourse during the one-year follow-up period. For a handful of women, this was accentuated if there had been a significant event (e.g., cancer recurrence), low sexual desire, pain during intercourse or a medical condition of the partner.

*“It helped me to understand how my body reacted to a lot of things, to understand that I was not alone and it helped me to accept myself and accept living my sex life in a different way. It [the treatment] allowed us to make different connections. There is a lot, really a lot of affection. It starts slowly, and, in the end, it becomes intense. This is what is new, this is what we learned.”–C17B*

*THEME 3*. *Reduction in urinary symptoms*. Half of the sample experienced either stress urinary incontinence, urgency urinary incontinence or symptoms of urinary urgency before the study and all women reported significant improvements following PFPT. Participants observed that the pelvic floor muscle exercises in addition to bladder training increased their muscle awareness, strength and endurance to activate their pelvic floor muscles when needed. For instance, it gave them the means to delay the urge to urinate or to hold the urine for longer periods.

*“Before, I used to go to the bathroom… a lot! Almost every hour, and now I go like three or four times a day and that’s enough. So, for sure, there is a difference there as well.”–C14*

*“I used to go to the bathroom all the time, all the time, and she [the physical therapist] gave me some tips for the bladder and exercises, and it’s getting better in that respect too.”–C111*

*“All the exercises, the squeezing and all that helped. You squeeze and it calms your bladder. I didn’t think it would work. Listen, I can even hold my urine when I go to the bathroom… Before, when I saw the toilet, I had to run and when I saw the toilet bowl, I leaked two or three drops. But now, I am able to hold it. I know what to do.” –C10*

Interestingly, two women said that having had painful urination and difficulty retaining high volumes of urine since the oncological treatments and they explained that, by releasing tensions in the pelvic area, the PFPT modalities such as manual therapy and auto-insertion exercises helped them to resolve these issues.

*“It was stiff near the bladder and it hurt. I felt the bladder was jammed, it was like there was no room for it to fill up. So, the physical therapy helped to relax the tensions and my bladder had more room so I needed to urinate less often. At night, I used to get up every three hours, I get up less now, so I sleep better. Everything is going in the right direction.”–C17B*

## Discussion

This mixed-method study provides evidence that the improvements in pain, sexual functioning, sexual distress, body image concerns, pain anxiety, pain catastrophizing, painful intercourse self-efficacy, depressive symptoms, urinary symptoms, vaginal symptoms and sexual matters following multimodal PFPT can be sustained at one-year follow-up in gynecological cancer survivors with dyspareunia. Furthermore, reduction in pain during sexual intercourse, improvement in sexual functioning and reduction in urinary symptoms were reported by participants as the most meaningful effects during the interviews. In addition, participants expressed these treatment effects in relation to adherence. They also emphasized that the treatment led to positive biological, psychological and social changes which contributed to the improvements in dyspareunia and sexual functioning.

This is the first study to examine whether the short-term improvements following multimodal PFPT are maintained over time in gynecological cancer survivors affected by dyspareunia [55]. Interventional studies conducted to date in women who had been treated for gynecological cancer were not specific to dyspareunia (e.g., urinary incontinence, vaginal atrophy or low sexual desire) [56–64]. To our knowledge, only a few cohort studies included a follow-up assessment beyond six months [60,62,65,66]. Improvement in sexual functioning have been seen following interventions integrating psychosexual education and unsupervised pelvic floor exercises in gynecological cancer survivors [60,62], which is consistent with the current study. However, their target population was different as women with or without symptoms were included immediately after oncological treatments. The experimental interventions were also designed to prevent or address common symptoms in gynecological cancer survivors while not specifically targeting dyspareunia [60,62]. In contrast, our sample was probably more affected at baseline as all women presented a pain intensity of more than 5 on the NRS for a median duration of three years, representing chronic moderate-to-severe dyspareunia [67,68]. Despite this chronicity and severity, it is noteworthy that participants still observed and reported sustained significant effects one year later.

The women in the present study expressed meaningful improvements in pain during intercourse, sexual functioning and urinary symptoms that lasted one year after PFPT. Similar findings were found in studies investigating multimodal PFPT effects in younger women suffering from vulvar pain with no history of cancer, although the available data is limited to a six-month follow-up in this population [69,70]. Morin et al. [70] in a large multicenter randomized controlled trial (n = 212) revealed reductions in pain and sexual distress with improved sexual functioning from baseline to six-month follow-up, compared to topical lidocaine, a frequent first-line treatment. Moreover, a recent Cochrane meta-analysis concluded that pelvic floor muscle training can reduce or cure urinary symptoms in women without a history of cancer [71], which is in line with our results. It is worth noting that the majority of studies conducted in women affected by dyspareunia with no history of cancer applied quantitative methods to evaluate the effects of multimodal PFPT [69,70,72]. A quantitative research design could only provide a narrow view of PFPT effects, as demonstrated in the current study.

Quantitative results combined with the participants’ inputs suggest that multimodal PFPT improved multiple dimensions of the biopsychological framework of dyspareunia [6,9,11,73], and these improvements remained at one-year follow-up. More precisely, the effects on pain during intercourse, sexual functioning and urinary symptoms were explained by gynecological cancer survivors through biological, psychological and social changes attributable to PFPT modalities. Gynecological cancer survivors emphasized the role of multimodal PFPT in the effects perceived and how it helped them to achieve pain-free sexual activities or improve their sexual functioning or behavior. It is notable that the treatment not only improved the pelvic floor tissues, as observed in short-term studies using objective tools [28,72,74], but also had a direct or indirect positive impact on psychological and social dimensions according to our cohort. Qualitative data suggested that performing PFPT exercices or having sexual intercourse regularly could be important to retain the biological changes related to pain for certain women. These details show that treatment effects over time could depend on adherence in the long term. Comparing our results to the studies conducted in women with no history of cancer, only two studies [75,76] to date have investigated the improvements following myofascial release techniques [76] and multimodal PFPT at three-month follow-up using a shorter interview [75] for dyspareunia in young women. The latter study reported similar effects in regard to muscle awareness, knowledge and communication about pain, self-efficacy, self-esteem, sexual confidence, attitudes about sexuality and relationship with the partner [75]. However, it should be underlined that our group of participants was still experiencing substantial effects at one-year follow-up after PFPT even though they had been treated for cancer, were older and had had dyspareunia for a median duration of three years. As opposed to previous work [75,76], our study is the first to triangulate data from different methods and to present extensively qualitative findings about multimodal PFPT effects by reporting the participants’ inputs that supported our interpretation while providing a deeper understanding. Overall, our findings suggest multimodal PFPT as a biopsychosocial treatment for reducing dyspareunia and improving sexual functioning.

The main strength of this study is the integration of quantitative and qualitative methods to allow data triangulation and complementarity to fully capture the treatment effects [77–79]. Validated scales and questionnaires were used to assess the quantitative outcomes. Intention-to-treat analyses were conducted and considered multiple comparisons as well as missing data. The high participation rate in qualitative interviews promoted a wide range of perspectives and shed light on how multimodal PFPT could have influenced dimensions other than the well-known biological dimension. The mixed-method design has allowed us to illustrate eloquently the quantitative findings supported by statistics and through the perceptions of women. Our results should, however, be interpreted within the context of certain limitations. The absence of a control group limits the causal inference. Nonetheless, the women’s perceptions support the role of PFPT in leading to these effects. They also did not attempt other treatments during the follow-up period. Moreover, it is unlikely that they would have improved without any treatment, given that they were suffering from dyspareunia for a median time of approximately three years and that sexual issues tend to persist over time [80,81]. Even though these aspects are suggestive of a causal inference of PFPT on outcomes, a randomized controlled trial is ultimately required to confirm the long-term efficacy of this treatment. As the PFPT treatment combined multiple modalities, it is difficult to isolate their respective effect on the outcomes. Moreover, determining precisely how the treatment effects (i.e., reduction in pain during intercourse, improvement in sexual functioning and reduction in urinary symptoms) and their modulating and contributing factors (i.e., adherence as well as biological, psychological and social changes) interacted was not feasible. It is worth mentioning that it has frequently been reported that these may overlap and influence each other dynamically and differently among gynecological cancer survivors [18,82,83]. A biopsychosocial treatment approach could have contributed to the magnitude of the effects [26].

## Conclusions

Findings of this one-year follow-up mixed-method study suggest that the short-term improvements in pain during sexual intercourse, sexual functioning and urinary incontinence following PFPT can be sustained over time in gynecological cancer survivors with dyspareunia. Although a randomized controlled trial is still required to confirm the efficacy, multimodal PFPT showed beneficial effects of treating dyspareunia in this population through biological, psychological and social changes after one year. The study therefore supports the biopsychosocial role of multimodal PFPT in gynecological cancer survivors who are frequently affected by pain and other types of sexual dysfunction. This treatment could be implemented in multidisciplinary cancer care.

## Supporting information

S1 File Semi-structured interview guide. (DOCX)

## References

[1] Howlader N, Noone AM, Krapcho M, Miller D, Brest A, Yu M, et al. SEER Cancer Statistics Review, 1975–2017, National Cancer Institute. Bethesda, 2020.

[2] Torre LA, Islami F, Siegel RL, Ward EM, Jemal A. Global cancer in women: burden and trends. Cancer Epidemiol Biomarkers Prev. 2017;26:444–57. doi: 10.1158/1055-9965.EPI-16-0858 28223433

[3] Rutledge TL, Heckman SR, Qualls C, Muller CY, Rogers RG. Pelvic floor disorders and sexual function in gynecologic cancer survivors: a cohort study. Am J Obstet Gynecol. 2010;203:514 e1–7. doi: 10.1016/j.ajog.2010.08.004 20869691 PMC5356373

[4] Stinesen Kollberg K, Waldenstrom AC, Bergmark K, Dunberger G, Rossander A, Wilderang U, et al. Reduced vaginal elasticity, reduced lubrication, and deep and superficial dyspareunia in irradiated gynecological cancer survivors. Acta Oncol. 2015;54:772–9. doi: 10.3109/0284186X.2014.1001036 25761090

[5] Coady D, Kennedy V. Sexual health in women affected by cancer: focus on sexual pain. Obstet Gynecol. 2016;128:775–91. doi: 10.1097/AOG.0000000000001621 27607852

[6] Bergeron S, Corsini-Munt S, Aerts L, Rancourt K, Rosen NO. Female sexual pain disorders: a review of the literature on etiology and treatment. Curr Sex Health Rep. 2015;7:159–69.

[7] Engel GL. The need for a new medical model: a challenge for biomedicine. Science. 1977;196:129–36. doi: 10.1126/science.847460 847460

[8] Cyr MP, Dumoulin C, Bessette P, Pina A, Gotlieb WH, Lapointe-Milot K, et al. Characterizing pelvic floor muscle function and morphometry in survivors of gynecological cancer who have dyspareunia: a comparative cross-sectional study. Phys Ther. 2021;101. doi: 10.1093/ptj/pzab042 33522584

[9] Thomtén J, Linton SJ. A psychological view of sexual pain among women: applying the fear-avoidance model. Women’s health (London, England). 2013;9:251–63. doi: 10.2217/whe.13.19 23638781

[10] Lemieux AJ, Bergeron S, Steben M, Lambert B. Do romantic partners’ responses to entry dyspareunia affect women’s experience of pain? The roles of catastrophizing and self-efficacy. J Sex Med. 2013;10:2274–84. doi: 10.1111/jsm.12252 23809759

[11] Corsini-Munt S, Rancourt KM, Dubé JP, Rossi MA, Rosen NO. Vulvodynia: a consideration of clinical and methodological research challenges and recommended solutions. J Pain Res. 2017;10:2425–36. doi: 10.2147/JPR.S126259 29070953 PMC5640408

[12] Juraskova I, Butow P, Robertson R, Sharpe L, McLeod C, Hacker N. Post-treatment sexual adjustment following cervical and endometrial cancer: a qualitative insight. Psychooncology. 2003;12:267–79. doi: 10.1002/pon.639 12673810

[13] Cleary V, Hegarty J. Understanding sexuality in women with gynaecological cancer. Eur J Oncol Nurs. 2011;15:38–45. doi: 10.1016/j.ejon.2010.05.008 20584629

[14] Bowes H, Jones G, Thompson J, Alazzam M, Wood H, Hinchliff S, et al. Understanding the impact of the treatment pathway upon the health-related quality of life of women with newly diagnosed endometrial cancer—a qualitative study. Eur J Oncol Nurs. 2014;18:211–7. doi: 10.1016/j.ejon.2013.10.007 24290535

[15] Reis N, Beji NK, Coskun A. Quality of life and sexual functioning in gynecological cancer patients: results from quantitative and qualitative data. Eur J Oncol Nurs. 2010;14:137–46. doi: 10.1016/j.ejon.2009.09.004 19836305

[16] Sekse RJ, Raaheim M, Blaaka G, Gjengedal E. Life beyond cancer: women’s experiences 5 years after treatment for gynaecological cancer. Scand J Caring Sci. 2010;24:799–807. doi: 10.1111/j.1471-6712.2010.00778.x 20487404

[17] Vermeer WM, Bakker RM, Kenter GG, Stiggelbout AM, Ter Kuile MM. Cervical cancer survivors’ and partners’ experiences with sexual dysfunction and psychosexual support. Support Care Cancer. 2016;24:1679–87. doi: 10.1007/s00520-015-2925-0 26412245 PMC4766206

[18] Bakker RM, Kenter GG, Creutzberg CL, Stiggelbout AM, Derks M, Mingelen W, et al. Sexual distress and associated factors among cervical cancer survivors: a cross-sectional multicenter observational study. Psychooncology. 2017;26:1470–7. doi: 10.1002/pon.4317 27862635

[19] Stabile C, Gunn A, Sonoda Y, Carter J. Emotional and sexual concerns in women undergoing pelvic surgery and associated treatment for gynecologic cancer. Transl Androl Urol. 2015;4:169–85. doi: 10.3978/j.issn.2223-4683.2015.04.03 26816823 PMC4708131

[20] Abbott-Anderson K, Kwekkeboom KL. A systematic review of sexual concerns reported by gynecological cancer survivors. Gynecol Oncol. 2012;124:477–89. doi: 10.1016/j.ygyno.2011.11.030 22134375

[21] Izycki D, Wozniak K, Izycka N. Consequences of gynecological cancer in patients and their partners from the sexual and psychological perspective. Prz Menopauzalny. 2016;15:112–6. doi: 10.5114/pm.2016.61194 27582686 PMC4993986

[22] Carter J, Lacchetti C, Andersen BL, Barton DL, Bolte S, Damast S, et al. Interventions to address sexual problems in people with cancer: American Society of Clinical Oncology Clinical Practice Guideline Adaptation of Cancer Care Ontario Guideline. J Clin Oncol. 2018;36:492–511. doi: 10.1200/JCO.2017.75.8995 29227723

[23] Huffman LB, Hartenbach EM, Carter J, Rash JK, Kushner DM. Maintaining sexual health throughout gynecologic cancer survivorship: a comprehensive review and clinical guide. Gynecol Oncol. 2016;140:359–68. doi: 10.1016/j.ygyno.2015.11.010 26556768 PMC4835814

[24] Crean-Tate KK, Faubion SS, Pederson HJ, Vencill JA, Batur P. Management of genitourinary syndrome of menopause in female cancer patients: a focus on vaginal hormonal therapy. Am J Obstet Gynecol. 2020;222:103–13. doi: 10.1016/j.ajog.2019.08.043 31473229

[25] Morin M, Carroll MS, Bergeron S. Systematic review of the effectiveness of physical therapy modalities in women with provoked vestibulodynia. Sex Med Rev. 2017;5:295–322. doi: 10.1016/j.sxmr.2017.02.003 28363763

[26] Wijma AJ, van Wilgen CP, Meeus M, Nijs J. Clinical biopsychosocial physiotherapy assessment of patients with chronic pain: the first step in pain neuroscience education. Physiother Theory Pract. 2016;32:368–84. doi: 10.1080/09593985.2016.1194651 27351769

[27] Cyr MP, Dumoulin C, Bessette P, Pina A, Gotlieb WH, Lapointe-Milot K, et al. Feasibility, acceptability and effects of multimodal pelvic floor physical therapy for gynecological cancer survivors suffering from painful sexual intercourse: a multicenter prospective interventional study. Gynecol Oncol. 2020;159:778–84. doi: 10.1016/j.ygyno.2020.09.001 33010968

[28] Cyr MP, Dumoulin C, Bessette P, Pina A, Gotlieb WH, Lapointe-Milot K, et al. Changes in pelvic floor morphometry and muscle function after multimodal physiotherapy for gynaecological cancer survivors suffering from dyspareunia: a prospective interventional study. Physiotherapy. 2021;In press. 10.1016/j.physio.2021.09.003 35093737

[29] Cyr MP, Dumoulin C, Bessette P, Pina A, Gotlieb WH, Lapointe-Milot K, et al. A prospective single-arm study evaluating the effects of a multimodal physical therapy intervention on psychosexual outcomes in women with dyspareunia after gynecologic cancer. J Sex Med. 2021;18:946–54. doi: 10.1016/j.jsxm.2021.02.014 33931347

[30] Hlatky MA, Owens DK, Sanders GD. Cost-effectiveness as an outcome in randomized clinical trials. Clinical trials (London, England). 2006;3:543–51. doi: 10.1177/1740774506073105 17170039

[31] Edmunds K, Ling R, Shakeshaft A, Doran C, Searles A. Systematic review of economic evaluations of interventions for high risk young people. BMC Health Serv Res. 2018;18:660. doi: 10.1186/s12913-018-3450-x 30139384 PMC6108123

[32] Fitzpatrick T, Perrier L, Shakik S, Cairncross Z, Tricco AC, Lix L, et al. Assessment of long-term follow-up of randomized trial participants by linkage to routinely collected data: a scoping review and analysis. JAMA. 2018;1:e186019-e. doi: 10.1001/jamanetworkopen.2018.6019 30646311 PMC6324362

[33] Frawley HC, Dean SG, Slade SC, Hay-Smith EJC. Is pelvic-floor muscle training a physical therapy or a behavioral therapy? A call to name and report the physical, cognitive, and behavioral elements. Phys Ther. 2017;97:425–37. doi: 10.1093/ptj/pzx006 28499001

[34] Abildgaard JS, Saksvik PØ, Nielsen K. How to measure the intervention process? An assessment of qualitative and quantitative approaches to data collection in the process evaluation of organizational interventions. Front Psychol. 2016;7:1380. doi: 10.3389/fpsyg.2016.01380 27713707 PMC5031711

[35] Blackman T, Wistow J, Byrne D. Using qualitative comparative analysis to understand complex policy problems. Evaluation. 2013;19:126–40.

[36] Hartmann D, Strauhal MJ, Nelson CA. Treatment of women in the United States with localized, provoked vulvodynia: practice survey of women’s health physical therapists. J Reprod Med. 2007;52:48–52. 17286069

[37] Dworkin RH, Turk DC, Wyrwich KW, Beaton D, Cleeland CS, Farrar JT, et al. Interpreting the clinical importance of treatment outcomes in chronic pain clinical trials: IMMPACT recommendations. J Pain. 2008;9:105–21. doi: 10.1016/j.jpain.2007.09.005 18055266

[38] Melzack R. The McGill Pain Questionnaire: major properties and scoring methods. Pain. 1975;1:277–99. doi: 10.1016/0304-3959(75)90044-5 1235985

[39] Wiegel M, Meston C, Rosen R. The female sexual function index (FSFI): cross-validation and development of clinical cutoff scores. J Sex Marital Ther. 2005;31:1–20. doi: 10.1080/00926230590475206 15841702

[40] Meyer-Bahlburg HF, Dolezal C. The female sexual function index: a methodological critique and suggestions for improvement. J Sex Marital Ther. 2007;33:217–24. doi: 10.1080/00926230701267852 17454519

[41] Santos-Iglesias P, Mohamed B, Walker LM. A systematic review of sexual distress measures. J Sex Med. 2018;15:625–44. doi: 10.1016/j.jsxm.2018.02.020 29576431

[42] Derogatis L, Clayton A, Lewis-D’Agostino D, Wunderlich G, Fu Y. Validation of the female sexual distress scale-revised for assessing distress in women with hypoactive sexual desire disorder. J Sex Med. 2008;5:357–64. doi: 10.1111/j.1743-6109.2007.00672.x 18042215

[43] Hopwood P, Fletcher I, Lee A, Al Ghazal S. A body image scale for use with cancer patients. Eur J Cancer. 2001;37:189–97. doi: 10.1016/s0959-8049(00)00353-1 11166145

[44] McCracken LM, Dhingra L. A short version of the Pain Anxiety Symptoms Scale (PASS-20): preliminary development and validity. Pain Res Manag. 2002;7:45–50. doi: 10.1155/2002/517163 16231066

[45] Sullivan MJL, Bishop SR, Pivik J. The Pain Catastrophizing Scale: development and validation. Psychol Assess. 1995;7:524–32.

[46] Desrochers G, Bergeron S, Khalife S, Dupuis MJ, Jodoin M. Fear avoidance and self-efficacy in relation to pain and sexual impairment in women with provoked vestibulodynia. Clin J Pain. 2009;25:520–7. doi: 10.1097/AJP.0b013e31819976e3 19542801

[47] Avery K, Donovan J, Peters TJ, Shaw C, Gotoh M, Abrams P. ICIQ: a brief and robust measure for evaluating the symptoms and impact of urinary incontinence. Neurourol Urodyn. 2004;23:322–30. doi: 10.1002/nau.20041 15227649

[48] Price N, Jackson SR, Avery K, Brookes ST, Abrams P. Development and psychometric evaluation of the ICIQ Vaginal Symptoms Questionnaire: the ICIQ-VS. BJOG. 2006;113:700–12. doi: 10.1111/j.1471-0528.2006.00938.x 16709214

[49] Farrar JT, Young JP Jr., LaMoreaux L, Werth JL, Poole RM. Clinical importance of changes in chronic pain intensity measured on an 11-point numerical pain rating scale. Pain. 2001;94:149–58. doi: 10.1016/S0304-3959(01)00349-9 11690728

[50] Venegas M, Carrasco B, Casas-Cordero R. Factors influencing long-term adherence to pelvic floor exercises in women with urinary incontinence. Neurourol Urodyn. 2018;37:1120–7. doi: 10.1002/nau.23432 29095511

[51] Schielzeth H, Dingemanse NJ, Nakagawa S, Westneat DF, Allegue H, Teplitsky C, et al. Robustness of linear mixed-effects models to violations of distributional assumptions. Methods Ecol Evol. 2020;11:1141–52.

[52] Ashbeck EL, Bell ML. Single time point comparisons in longitudinal randomized controlled trials: power and bias in the presence of missing data. BMC Med Serv Res. 2016;16:43. doi: 10.1186/s12874-016-0144-0 27068578 PMC4828848

[53] Harrell FJ. Regression modeling strategies: with applications to linear models, logistic and ordinal regression, and survival analysis. 2nd ed. New York: Springer; 2015.

[54] Braun V, Clarke V. Using thematic analysis in psychology. Qualitative research in psychology. 2006;3:77–101.

[55] Brennen R, Lin KY, Denehy L, Frawley HC. The effect of pelvic floor muscle interventions on pelvic floor dysfunction after gynecological cancer treatment: a systematic review. Phys Ther. 2020;100:1357–71. doi: 10.1093/ptj/pzaa081 32367126

[56] Yang EJ, Lim JY, Rah UW, Kim YB. Effect of a pelvic floor muscle training program on gynecologic cancer survivors with pelvic floor dysfunction: a randomized controlled trial. Gynecol Oncol. 2012;125:705–11. doi: 10.1016/j.ygyno.2012.03.045 22472463

[57] Rutledge TL, Rogers R, Lee SJ, Muller CY. A pilot randomized control trial to evaluate pelvic floor muscle training for urinary incontinence among gynecologic cancer survivors. Gynecol Oncol. 2014;132:154–8. doi: 10.1016/j.ygyno.2013.10.024 24183730 PMC5399541

[58] Bober SL, Recklitis CJ, Michaud AL, Wright AA. Improvement in sexual function after ovarian cancer: effects of sexual therapy and rehabilitation after treatment for ovarian cancer. Cancer. 2018;124:176–82. doi: 10.1002/cncr.30976 28881456 PMC5734953

[59] Damast S, Jeffery DD, Son CH, Hasan Y, Carter J, Lindau ST, et al. Literature review of vaginal stenosis and dilator use in radiation oncology. Pract Radiat Oncol. 2019;9:479–91. doi: 10.1016/j.prro.2019.07.001 31302301 PMC7944435

[60] Bakker RM, Mens JW, de Groot HE, Tuijnman-Raasveld CC, Braat C, Hompus WC, et al. A nurse-led sexual rehabilitation intervention after radiotherapy for gynecological cancer. Support Care Cancer. 2017;25:729–37. doi: 10.1007/s00520-016-3453-2 27787681 PMC5266770

[61] Carter J, Goldfarb S, Baser RE, Goldfrank DJ, Seidel B, Milli L, et al. A single-arm clinical trial investigating the effectiveness of a non-hormonal, hyaluronic acid-based vaginal moisturizer in endometrial cancer survivors. Gynecol Oncol. 2020;158:366–74. doi: 10.1016/j.ygyno.2020.05.025 32522420 PMC7423634

[62] Carter J, Stabile C, Seidel B, Baser RE, Goldfarb S, Goldfrank DJ. Vaginal and sexual health treatment strategies within a female sexual medicine program for cancer patients and survivors. J Cancer Surviv. 2017;11:274–83. doi: 10.1007/s11764-016-0585-9 27868156 PMC5357589

[63] Li J, Huang J, Zhang J, Li Y. A home-based, nurse-led health program for postoperative patients with early-stage cervical cancer: a randomized controlled trial. Eur J Oncol Nurs. 2016;21:174–80. doi: 10.1016/j.ejon.2015.09.009 26482004

[64] Zhu G, Li X, Yang S. Effect of postoperative intervention on the quality of life of patients with cervical cancer. 2016;28:819–22.

[65] Bahng AY, Dagan A, Bruner DW, Lin LL. Determination of prognostic factors for vaginal mucosal toxicity associated with intravaginal high-dose rate brachytherapy in patients with endometrial cancer. Int J Radiat Oncol Biol Phys. 2012;82:667–73. doi: 10.1016/j.ijrobp.2010.10.071 21300451

[66] Gondi V, Bentzen SM, Sklenar KL, Dunn EF, Petereit DG, Tannehill SP, et al. Severe late toxicities following concomitant chemoradiotherapy compared to radiotherapy alone in cervical cancer: an inter-era analysis. Int J Radiat Oncol Biol Phys. 2012;84:973–82. doi: 10.1016/j.ijrobp.2012.01.064 22898381 PMC3706199

[67] Turner JA, Franklin G, Heagerty PJ, Wu R, Egan K, Fulton-Kehoe D, et al. The association between pain and disability. Pain. 2004;112:307–14. doi: 10.1016/j.pain.2004.09.010 15561386

[68] Dworkin RH, Turk DC, Peirce-Sandner S, Baron R, Bellamy N, Burke LB, et al. Research design considerations for confirmatory chronic pain clinical trials: IMMPACT recommendations. Pain. 2010;149:177–93. doi: 10.1016/j.pain.2010.02.018 20207481

[69] Goldfinger C, Pukall CF, Thibault-Gagnon S, McLean L, Chamberlain S. Effectiveness of cognitive-behavioral therapy and physical therapy for provoked vestibulodynia: a randomized pilot study. J Sex Med. 2016;13:88–94. doi: 10.1016/j.jsxm.2015.12.003 26755091

[70] Morin M, Dumoulin C, Bergeron S, Mayrand MH, Khalifé S, Waddell G, et al. Multimodal physical therapy versus topical lidocaine for provoked vestibulodynia: a multicenter, randomized trial. Am J Obstet Gynecol. 2021;224:189.e1–.e12. doi: 10.1016/j.ajog.2020.08.038 32818475

[71] Dumoulin C, Cacciari LP, Hay-Smith EJC. Pelvic floor muscle training versus no treatment, or inactive control treatments, for urinary incontinence in women. Cochrane Database Syst Rev. 2018;10:Cd005654. doi: 10.1002/14651858.CD005654.pub4 30288727 PMC6516955

[72] Gentilcore-Saulnier E, McLean L, Goldfinger C, Pukall CF, Chamberlain S. Pelvic floor muscle assessment outcomes in women with and without provoked vestibulodynia and the impact of a physical therapy program. J Sex Med. 2010;7:1003–22. doi: 10.1111/j.1743-6109.2009.01642.x 20059663

[73] Thomtén J, Lundahl R, Stigenberg K, Linton S. Fear avoidance and pain catastrophizing among women with sexual pain. Women’s health (London, England). 2014;10:571–81. doi: 10.2217/whe.14.51 25482484

[74] Mercier J, Morin M, Tang A, Reichetzer B, Lemieux MC, Samir K, et al. Pelvic floor muscle training: mechanisms of action for the improvement of genitourinary syndrome of menopause. Climacteric. 2020;23:468–73. doi: 10.1080/13697137.2020.1724942 32105155

[75] Goldfinger C, Pukall CF, Gentilcore-Saulnier E, McLean L, Chamberlain S. A prospective study of pelvic floor physical therapy: pain and psychosexual outcomes in provoked vestibulodynia. J Sex Med. 2009;6:1955–68. doi: 10.1111/j.1743-6109.2009.01304.x 19453890

[76] Mackenzie N. A phenomenological study of women who presented to a physiotherapy-led continence service with dyspareunia and were treated with trigger point massage. J Assoc Chart Physiother Women’s Health. 2009;105:24–39.

[77] Schoonenboom J, Johnson RB. How to construct a mixed methods research design. Kolner Z Soz Sozpsychol. 2017;69:107–31. doi: 10.1007/s11577-017-0454-1 28989188 PMC5602001

[78] Irvine FE, Clark MT, Efstathiou N, Herber OR, Howroyd F, Gratrix L, et al. The state of mixed methods research in nursing: a focused mapping review and synthesis. J Adv Nurs. 2020;76:2798–809. doi: 10.1111/jan.14479 32896959

[79] O’Cathain A, Murphy E, Nicholl J. The quality of mixed methods studies in health services research. J Health Serv Res Policy. 2008;13:92–8. doi: 10.1258/jhsrp.2007.007074 18416914

[80] Pieterse QD, Kenter GG, Maas CP, de Kroon CD, Creutzberg CL, Trimbos JB, et al. Self-reported sexual, bowel and bladder function in cervical cancer patients following different treatment modalities: longitudinal prospective cohort study. Int J Gynecol Cancer. 2013;23:1717–25. doi: 10.1097/IGC.0b013e3182a80a65 24172106

[81] DeSimone M, Spriggs E, Gass JS, Carson SA, Krychman ML, Dizon DS. Sexual dysfunction in female cancer survivors. Am J Clin Oncol. 2014;37:101–6. doi: 10.1097/COC.0b013e318248d89d 22643563

[82] Carpenter KM, Andersen BL, Fowler JM, Maxwell GL. Sexual self schema as a moderator of sexual and psychological outcomes for gynecologic cancer survivors. Arch Sex Behav. 2009;38:828–41. doi: 10.1007/s10508-008-9349-6 18418707 PMC2745514

[83] Andersen BL, Woods XA, Copeland LJ. Sexual self-schema and sexual morbidity among gynecologic cancer survivors. J Consult Clin Psychol. 1997;65:221–9. doi: 10.1037//0022-006x.65.2.221 9086685 PMC2705962

