# Supplementary material for: Improvements following multimodal pelvic floor physical therapy in gynecological cancer survivors suffering from pain during sexual intercourse: Results from a one-year follow-up mixed-method study
Source: PLoS One. 2022 Jan 25;17(1):e0262844. doi: 10.1371/journal.pone.0262844 (PMC8789131; doi:10.1371/journal.pone.0262844)
Supplement: S1 File — (DOCX) [file pone.0262844.s001.docx]

**Supporting information. S1 File. Semi-structured interview guide. [1]**

French version

Questions générales

1. Avez-vous perçu des changements après le traitement multimodal de physiothérapie que vous avez reçu?

a. Si oui, quels étaient-ils?

Déclencheurs : Changement sur la douleur? Changement sur la fonction sexuelle? Changement sur d’autres symptômes (p. ex., symptômes de problématiques pelvi-périnéales)? Changements physiques, psychologiques ou sociaux/relationnels?

b. Comment ces changements ont-ils évolués dans les 12 derniers mois?

Déclencheurs : Diriez-vous qu’ils se sont détériorés, améliorés ou maintenus?

c. Comment expliquez-vous ces changements?

Déclencheurs : À quoi les attribuez-vous?

Exemples de déclencheurs qui ont été utilisés pour obtenir plus d’information sur les perceptions des participantes quant aux effets du traitement :

1. Parlez-moi plus de [xxx].
2. Que voulez-vous dire lorsque vous dites [xxx]?
3. Décrivez-moi comment le traitement pourrait avoir mené à ce changement.

English version

General questions

1. Did you perceive any changes following the multimodal pelvic floor physical therapy treatment you received?

a. If so, what were they?

Probes: Changes in pain? Changes in sexual functioning? Changes in other symptoms (e.g., pelvic floor disorder symptoms)? Physical, psychological or social/relationship changes?

b. How did these changes evolved in the past 12 months?

Probes: Would you say they deteriorated, improved or maintained?

c. How do you explain these changes?

Probes: What do you attribute them to?

Examples of probes to obtain in-depth information about participants’ perceptions of treatment effects:

1. Tell me more about [xxx].
2. What do you mean when you say [xxx]?
3. Describe to me how the treatment could have led to this change.

[1] Cyr MP, Dostie R, Camden C, Dumoulin C, Bessette P, Pina A, et al. Improvements following multimodal pelvic floor physical therapy in gynecological cancer survivors suffering from pain during sexual intercourse: Results from a one-year follow-up mixed-method study. PLoS One. 2022.
